# Tall Cell Carcinoma With Reversed Polarity of the Breast

**DOI:** 10.7759/cureus.16814

**Published:** 2021-08-01

**Authors:** Gustavo Matute, Linda Barcenas, Carolina Bautista, Carlos Alberto Restrepo Ramirez, Nestor Llinas Quintero

**Affiliations:** 1 Pathology, Clinica Medellin, Medellin, COL; 2 Pathology and Laboratory Medicine, National University of Colombia, Bogotá, COL; 3 Pathology, Fundación Universitaria Ciencias de la Salud, Bogotá, COL; 4 Breast Surgery, Clinica Medellin, Medellín, COL; 5 Oncology, Fundación Colombiana de Cancerología - Clínica Vida., Medellín, COL

**Keywords:** triple negative breast neoplasms, cell polarity, breast cancer, breast cancer pathology, breast papillary lesions

## Abstract

Tall cell carcinoma with reversed polarity (TCCRP) of the breast is a rare entity with low potential for malignancy that exhibits some morphological similarities to the tall cell variant of papillary thyroid carcinoma. Immunohistochemical and molecular studies help establish the mammary origin of this neoplasm. Here, we describe the case of a 63-year-old woman with a finding of a nodular lesion during a screening mammogram, whose morphological findings and immunohistochemical studies confirmed the diagnosis of papillary high cell carcinoma with the reverse polarity of the mammary gland.

## Introduction

Tall cell carcinoma with reverse polarity (TCCRP) of the breast is a rare subtype of carcinoma with low systemic dissemination potential. A primary breast tumor similar to thyroid tall cell papillary carcinoma was first described in 2003 [1]. It exhibits typical histologic features such as solid papillary architecture, columnar morphology, granular eosinophilic cytoplasm, nuclear grooves, and pseudo-inclusions [2]. It was recently included as a new entity on the fifth edition of the WHO classification of tumors of the breast [1]. It has a particular immunohistochemistry profile that allows distinction from metastasis of thyroid carcinoma; additionally, it has specific mutations in the genes for the isocitrate dehydrogenase 2 (IDH2) and the catalytic subunit of the phosphatidylinositol 3 kinase (PIK3) [3]. Few cases have been reported of this entity. We present the case of a 63-year-old female patient and correlate our findings with previously reported cases in the literature.

## Case presentation

A 63-year-old female patient presented with a family history of breast cancer in two of her sisters, diagnosed at 63 and 57 years old, and a maternal aunt. Screening mammography showed focal asymmetry with a nodular appearance between the lower quadrants of the left breast, Breast Imaging Reporting and Data System (BI-RADS) 4c [4]. There were no nodules or other masses palpated on the physical exam of the breasts and cervical, axillary, or supraclavicular regions bilaterally. Breast ultrasonography showed a 10.7 x 8 mm isoechoic node with angulated borders at a six-hour position and 1 cm from the nipple. The biopsy of this lesion reported atypical ductal hyperplasia, which was followed by a lumpectomy.

**Figure 1:**
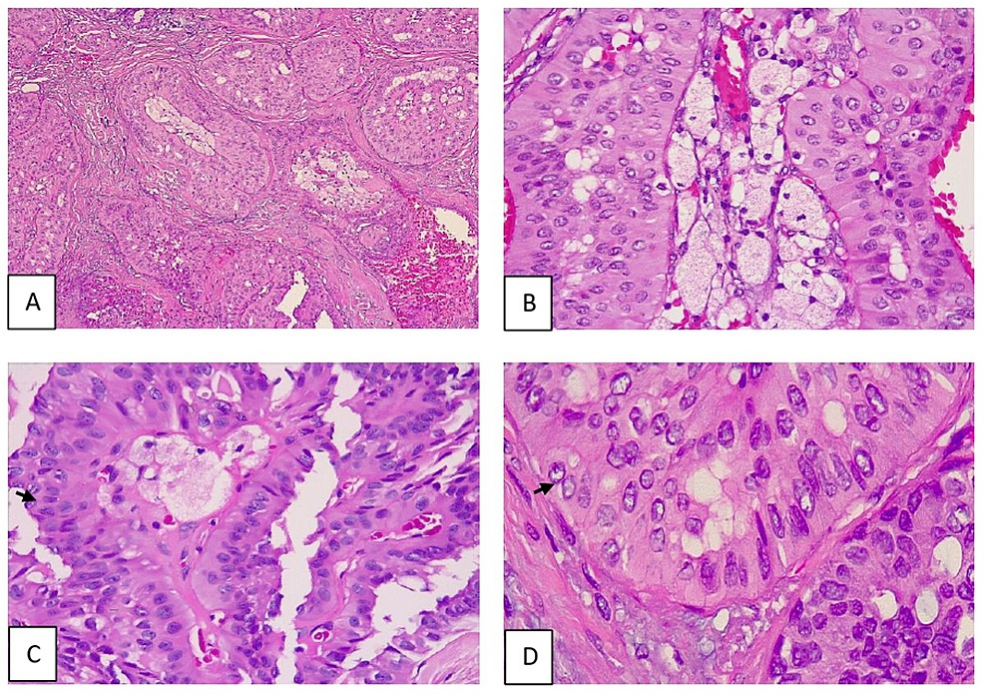
Microscopic findings (A) Neoplastic lesion composed of nests in various patterns: solid, papillary, and cribriform (H&E, x40). (B) Columnar cells with eosinophilic cytoplasm, apical oval-shaped nuclei surrounding fibrovascular cores with numerous foamy histiocytes (H&E, x100). (C, D) Some nuclei showed grooves and clearing (arrows) (H&E, x100 and x400).

On histological examination, malignant neoplasia was identified, constituted by trabeculae and nests made from large columnar cells with a wide granular cytoplasm. The nuclei were uniform, apical, oval, and round-shaped, some had grooves and prominent nucleoli; the nests had fibrovascular cores with numerous histiocytes (Figures 1A-1D). Three mitoses per high power field were identified and there was a poor inflammatory response. Foci of low-grade ductal carcinoma in situ with the cribriform pattern were identified in the periphery of the lesion but the margins were not compromised.

**Figure 2:**
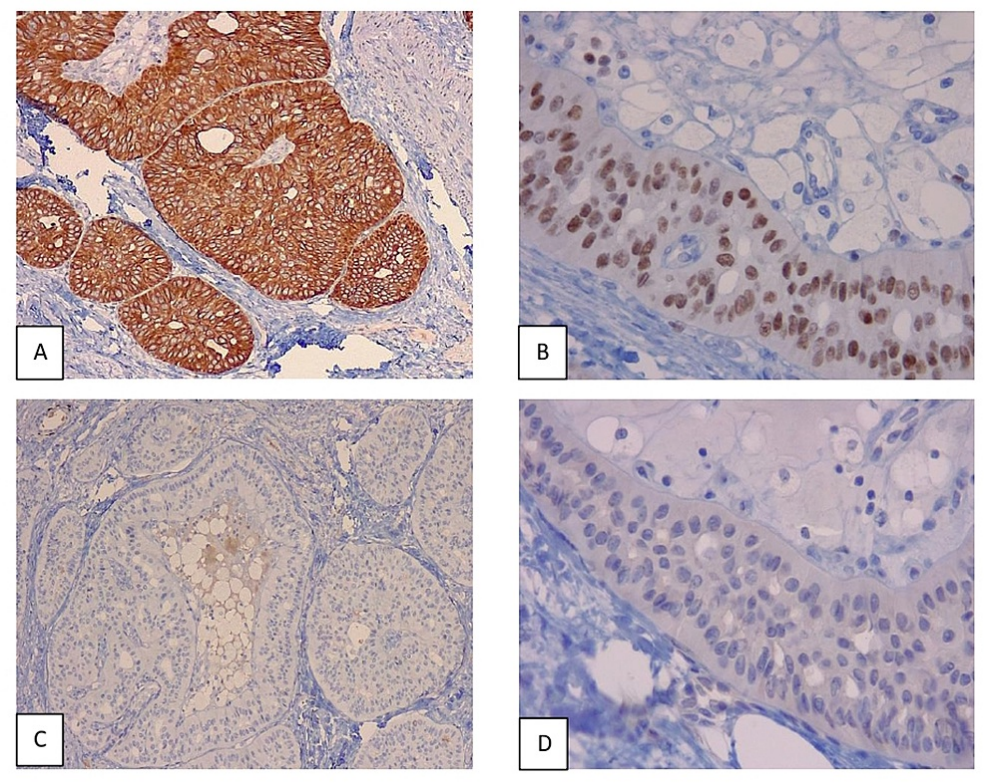
Immunohistochemistry studies (A, B) Tumor positive for CK5/6 (x100) and GATA 3 (x400). (C) The absence of myoepithelial cells inside and around tumoral nests (p63 x 100). (D) Tumoral cells negative for thyroglobin (x400).

Immunohistochemistry studies showed positivity to CK5/6, CK7, mammaglobin, gross cystic disease fluid protein-15 (GCDFP-15), and GATA binding protein 3 (GATA3). CK20, thyroid transcription factor-1 (TTF1), thyroglobin, and napsin-A were negative. Additionally, estrogen receptor (ER), androgen receptor (AR), progesterone receptor (PR), and Human epidermal growth factor receptor (HER2) were also negative. The cellular proliferation index (Ki67) was 2%; no myoepithelial cells were visualized in or around the tumor with the p63 and Monocyte chemotactic protein (MCP) markers (Figures 2A-2D). These findings were conclusive for infiltrative tall cell carcinoma of the breast with reverse polarity. Sentinel node biopsy and CT scan of the chest and abdomen were performed a month later; all of them showed no metastasis.

## Discussion

TCCRP is an uncommon entity, first reported in 2003 by Eusebi et al. [1]. Cases only occur in women ages 39-89 with a mean of 64 years at the time of diagnosis [5,6], usually found by palpation or by screening mammography. Size ranges from 6 to 42 mm [1,7-9] - this characteristic agrees with our case. Some patients have a prior history of contralateral breast carcinoma [10,11]. Generally speaking, TCCRP has an indolent biological behavior, with low metastatic potential, very few cases have reported regional, distant ganglionic dissemination or local recurrence, which is why it has an excellent prognosis in most cases [1,9,11,12]. In our index case here, metastases to regional lymph nodes were ruled out by a negative sentinel node and extended imaging studies revealed no distant metastases; the patient received primary conservative surgical management (lumpectomy) with full resection. A multidisciplinary team considered this case to be a rare entity with a good prognosis and favorable clinical outcome with no indication of benefit from systemic adjuvant chemotherapy, locoregional radiotherapy was recommended due to prior conservative surgery.

The WHO defines TCCRP as an invasive breast carcinoma characterized by tall columnar cells with a reverse polarity that form solid or papillary structures. It is frequently associated with hot spot mutations in residue 172 (R172) of the isocitrate dehydrogenase 2 (IDH2) gene [5]. Morphologically it is characterized by cuboidal or columnar oxyntic cells with abundant eosinophilic cytoplasm forming solid nests, with papillary or follicular architecture and separated by fibrous bands, with colloid-like secretion and the absence of myoepithelial cells [1,2,10]. Nuclei are located apically (reverse polarity) and may have optical clearing, grooves, and pseudo inclusions [1,9]. These features in addition to the presence of histiocytes are highly specific characteristics of TCCRP in this case.

Important differential diagnosis includes metastatic tall cell papillary carcinoma of the thyroid and other papillary lesions of the breast which is why a thorough examination of the H&E and immunochemistry studies is key [9]. Immunohistochemistry generally shows intense reactivity for the low weight (CK7) and high weigh cytokeratins (CK5/6, CK34ßE12) [1]. Typically, the expression of ER and PR are negative or weakly positive in 1%-10% of tumoral cells, HER-2 is negative [2,11]. Absent or weak expression for AR has also been reported in some patients [1,10]. They are considered low or intermediate-grade tumors with a low proliferation index (Ki67 < 10%) [2]. This distinction is important as the breast is a site for metastasis and up to 5% of them can be a thyroid carcinoma [13]. In this patient, negative immunohistochemistry for TTF1, thyroglobulin, and napsin-A confirms primary neoplasia of the breast and rule out a metastatic papillary carcinoma of the thyroid.

TCCRP is characterized by recurrent somatic mutations in the IDH2 R172, most of them R172S or R172T mutations [10] as well as somatic mutations that inactivate ten-eleven translocation 2 (TET2) [10]. Both can present concurrently with genes affecting the phosphatidylinositol 3-kinase (PI3K) pathway, there is even a secondary genotype-phenotype correlation [2,8,10,12]. Other mutations in genes such as ataxia telangiectasia mutated (ATM), KIT, and mesenchymal-epithelial transition factor (MET) have also been reported [8,12]. These and other alterations are different from those found in solid papillary carcinoma of the breast and papillary carcinoma of the thyroid [13]. In this case, although PCR and IHD2 sequencing were negative the histopathological characteristics were sufficient for the diagnosis of TCCRP.

## Conclusions

In conclusion, TCCRP is an infrequent clinical entity recently included in the WHO classification, with a triple-negative expression, low proliferation index, and indolent behavior in most cases. It presents with distinctive histological, immunohistochemical, and molecular characteristics; a high degree of certainty is required to avoid diagnostic pitfalls with other papillary or metastatic lesions of the breast, keeping in mind that a precise diagnosis is conducive to adequate treatment.
